# Seed-Based Biclustering of Gene Expression Data

**DOI:** 10.1371/journal.pone.0042431

**Published:** 2012-08-03

**Authors:** Jiyuan An, Alan Wee-Chung Liew, Colleen C. Nelson

**Affiliations:** 1 Institute of Health and Biomedical Innovation, Queensland University of Technology, Brisbane, Australia; 2 School of Information and Communication Technology, Gold Coast Campus, Griffith University, Queensland, Australia; Northwestern University Feinberg School of Medicine, United States of America

## Abstract

**Background:**

Accumulated biological research outcomes show that biological functions do not depend on individual genes, but on complex gene networks. Microarray data are widely used to cluster genes according to their expression levels across experimental conditions. However, functionally related genes generally do not show coherent expression across all conditions since any given cellular process is active only under a subset of conditions. Biclustering finds gene clusters that have similar expression levels across a subset of conditions. This paper proposes a seed-based algorithm that identifies coherent genes in an exhaustive, but efficient manner.

**Methods:**

In order to find the biclusters in a gene expression dataset, we exhaustively select combinations of genes and conditions as seeds to create candidate bicluster tables. The tables have two columns (a) a gene set, and (b) the conditions on which the gene set have dissimilar expression levels to the seed. First, the genes with less than the maximum number of dissimilar conditions are identified and a table of these genes is created. Second, the rows that have the same dissimilar conditions are grouped together. Third, the table is sorted in ascending order based on the number of dissimilar conditions. Finally, beginning with the first row of the table, a test is run repeatedly to determine whether the cardinality of the gene set in the row is greater than the minimum threshold number of genes in a bicluster. If so, a bicluster is outputted and the corresponding row is removed from the table. Repeating this process, all biclusters in the table are systematically identified until the table becomes empty.

**Conclusions:**

This paper presents a novel biclustering algorithm for the identification of additive biclusters. Since it involves exhaustively testing combinations of genes and conditions, the additive biclusters can be found more readily.

## Introduction

Gene expression level fluctuates across a set of conditions (or time points). The mechanism of gene regulation is complex at the molecular level; it is not a single gene, but many genes that simultaneously interact with each other to perform a biological function. Finding genes with similar behaviours in expression across a set of time points or conditions is the first and essential step. Microarray is a widely used technology to obtain gene expression levels for cell lines or tissues. The mining of microarray data constitutes an area of growing interest in the bioinformatics field. Clustering is an effective method used in microarray data analysis to reveal the mechanism of gene regulation for genetic diseases. Clustered genes have similar expression fluctuation across all conditions. However, since some diseases are only affected by a subset of conditions, it becomes necessary to identify those gene clusters that have a similar expression fluctuation across a specific subset of conditions; rather than identifying genes that have similar expression fluctuations across *all* experimental conditions. Biclustering [1], [2] describes the process by which a group of genes (rows) coherent within a group of conditions (columns) is identified. However, exhaustively evaluating all possible biclusters in a dataset is an NP-hard problem [3], [4], [5], where the main challenge lies in finding a way to efficiently select a subset of genes and conditions that satisfy the criterion of coherencies, especially when the numbers of genes and conditions/time points are large.

### Aims

Microarray data biclustering generally involves the analysis of very large datasets. Although many biclustering algorithms have been proposed [1], [2], [6], [7], [8], [9], [10], [11], [12], [13], there is still no efficient algorithm that can deal with very large microarray datasets. In this paper, a seed-based biclustering algorithm that identifies biclusters of coherent genes in an exhaustive, but efficient, manner is proposed.

Although there are several types of bicluster [9], the focus of this study is on the additive bicluster, which is the most common. An *additive bicluster* is the set of genes that have similar expression fluctuations in a subset of conditions. These genes could, for example, be regulated by common transcription factors or other chemical components, such as microRNA or other long non-coding RNA. This research could provide an effective tool, which would, for example, be used to assist biologists in the identification of regulation factors for certain diseases.

### Existing Algorithms

Cheng and Church [1] were the first to introduce biclustering into gene expression data. They introduced H-Score as a measure of the degree of coherence of a bicluster. The H-Score represents the variance of a particular subset of genes under a particular subset of conditions or time points. The central idea is to find biclusters whose H-score is less than a given threshold value δ.

One of the main problems with the δ-bicluster of Cheng and Church is that a submatrix of a δ-bicluster is not necessarily also a δ-bicluster, since the H-score is an averaged measurement of coherence in a δ-bicluster [14]. This results in a large number of false positives in the algorithm. Moreover, it does not perform an exhaustive search of all biclusters in the dataset.

Another family of biclustering algorithms is the geometric-based bicluster [2], [8], [12]. In this case, every gene is represented as a point in a high dimensional space. Biclusters are identified by finding points located in a hyper-plane. These algorithms are time and memory intensive for high dimensional data.

## Materials and Methods

Gene expressions can be illustrated as a profile whose vertical axis shows the expression level and whose horizontal axis represents conditions. Additive biclustered genes have similar trends across a certain number of conditions. If the profiles are displaced vertically by the appropriate amount, then all genes in a bicluster would have similar value across the conditions, and the additive biclustering problem becomes that of finding “biclusters with constant values on columns” [9]. Hence, additive biclustering is simplified to a process of finding the vertical-displacement-length for each gene. For different biclusters, every gene has a different vertical-displacement-length.

In this work, additive biclusters are identified based on “seeds”. All conditions in all genes are the potential seeds. All seeds are exhaustively tested to find biclusters that meet the criteria given by end-users. The following sections describe the details of this seed-based biclustering algorithm.

Table 1 shows the notations for a given additive bicluster that will be used in this paper.

**Table 1:** Notation.

E_gs_	Expression level of gene g in condition s
G	Genes in an additive bicluster
Σ	Selected conditions in a bicluster
Τ	A condition or time point
g_s_	The seed gene in a bicluster
s_s_	The seed condition in a bicluster
Δ	Maximum difference between genes in a bicluster
E	User-defined max difference between genes and seed gene
e	Threshold of relative expression level in a bicluster
τ	Threshold for filtering out un-interesting gene expression profile
min_gene	User-defined minimum number of genes in a bicluster
min_coherent_condition	User-defined minimum number of coherent conditions in a bicluster

The following sections describe the details of this seed-based biclustering algorithm:

It is assumed that if a pair of genes *g_a_* and *g_b_* are listed in a bicluster and τ is one selected condition, there exists a constant *C* such that:


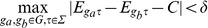
(1)

The constant *C* differs for different pairs of genes in a bicluster. If one gene is fixed and an appropriate constant added to every other gene in a bicluster, all genes in the bicluster would have similar expression levels across a common subset of conditions. As a result, the problem of finding additive biclusters becomes that of finding a suitable constant *C* for every gene in a given bicluster.

In this work, biclusters are identified through the use of “seed” where a “seed” is the combination of a gene and a condition. The gene and condition are called the *seed gene* and *seed condition,* respectively. The constant *C* of a gene *g* is the distance between gene *g* and the seed gene on the seed condition s_s_:


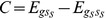
(2)

This method exhaustively enumerates Eq. (2) at all conditions to obtain the constant *C* that minimizes the maximum distance between the two genes.

**Figure 1:**
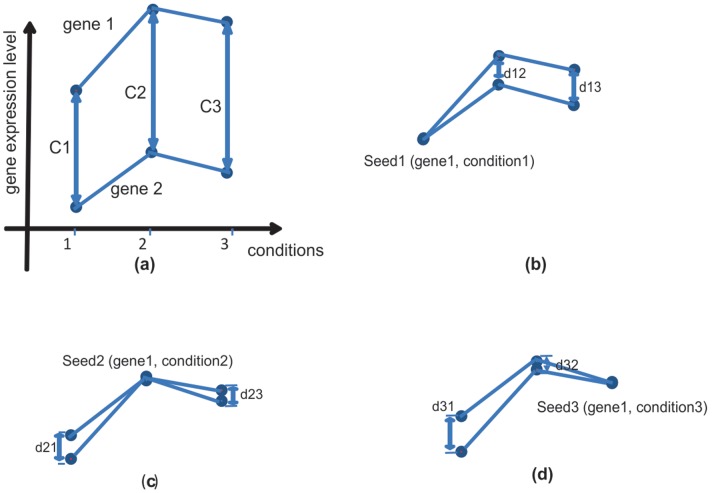
An example of finding suitable constant C that satisfies Eq (1). (a) Both gene1 and gene2 are measured by their expressed levels on three conditions. The distances between gene1 and gene2 on conditions 1, 2, and 3 are represented by C1, C2, and C3 respectively. (b) d_12_ and d_13_ are the distances between gene1 and gene2 on conditions 2 and 3 in terms of seed1. (c) and (d) show the distances of the two genes in term of seed2 and seed3.

Figure 1 shows the enumeration of *C* at three conditions 1, 2, and 3. If gene1 is selected as seed gene, then seed1, seed2, and seed3 correspond to conditions 1, 2 and 3 respectively. If gene 2 is set the constant value C for seed1, (i.e. C = C1), then gene 2 is displaced as shown in Figure 1 (b). The distance for gene 2 with respect to gene 1 in condition 2 and 3 are expressed as d_12_ = C2−C1 and d_13_ = C3−C1, respectively. Analogously, the distance for gene 2 for seed2 and seed3 can be expressed in Figure 1 (c) and (d) respectively. The results shows clearly that seed2 is the best seed for gene 1 and gene 2 in conditions 1, 2 and 3 because the smallest maximum distance between the two genes is d_21_ (d_21_<d_13_ and d_21_<d_31_). If the threshold for coherence is d_21_, the two genes are coherent in conditions 1 to 3. However, for seed2 and seed3, the two genes are not found to be coherent in other conditions. For the purpose of this study, the distance *d_i,j_* above is defined as the *relative value.* These relative values are used to find which conditions are coherent with respect to the seed.

**Figure 2:**
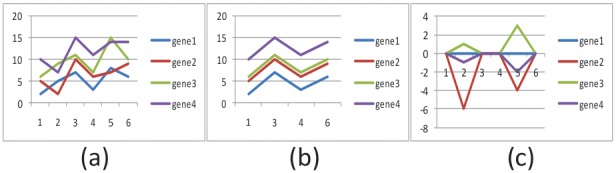
A simple example of, (a) Expression level across all conditions. (b) Expression level in conditions (1, 3, 4, 6) (c) Relative expression level across all conditions. Coherent conditions have small relative expression level, while non-coherent conditions have large relative expression level.

Figure 2(a) shows a very simple data set, which includes four genes (gene 1, gene 2, gene 3 and gene 4). The expression levels of the six conditions are shown on the vertical axis. The genes are not coherent over all conditions. However, with condition 1, 3, 4 and 6, the four genes have a coherent expression level as shown in Figure 2(b). Figure 2(c) shows the relative expression levels for this simple data set. With conditions 2 and 5, the relative expression level is far from zero, while with the 1, 3, 4 and 6 conditions, the relative expression level is zero, which means these four genes are strongly coherent with conditions 1, 3, 4 and 6 as shown in Figure 2(b).

**Figure 3:**
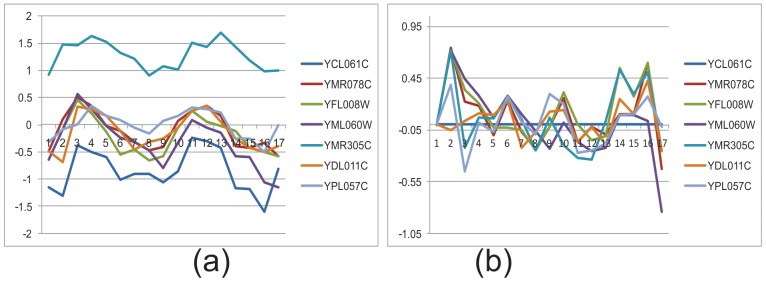
Gene expression level in absolute scale (a) and in relative scale (b). Vertical axis represents expression level normalized by z-score. Horizontal axis represents conditions or time points (1–17).

Figure 3 shows a bicluster taken from the real data of yeast cell cycle. The bicluster has seven genes: YCL061C, YMR078C, YFL008W, YML060W, YMR305C, YDL011C and YPL057C. The gene expression level (normalized by z-score) across all 17 time points is shown in Figure 3 (a). Several time points, such as 2 and 17, do not show coherent behaviour with the rest of the time points. If the first gene (YCL061C) is selected as seed gene and the first time point as seed condition, the relative expression values for all genes across all time points is obtained as shown in Figure 3 (b). Time points 2, 3, 14, 16 and 17 show a large deviation from zero.

**Figure 4:**
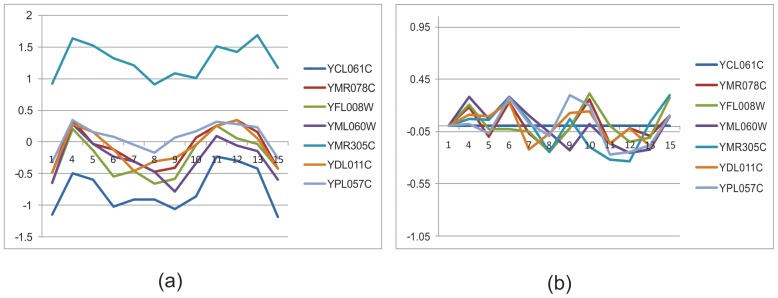
Gene expression level in absolute scale (a) and relative scale (b) after removing non-coherent time points.

If the error threshold of relative expression level is set to 0.35, these five time points are removed. Figure 4 (b) shows the time points where the relative values are less than 0.35, and the corresponding absolute expression levels are shown in Figure 4 (a). The genes are much more coherent in this subset of time points.

A seed gene g*_s_* is denoted as having gene expression level Eg_s_τ (*τ = 1,2,…,n*), where *τ* is a time point or condition. If time point, s_s_, is selected as a seed condition, for a given gene g_a_ whose expression level is Eg_a_τ, the relative expression level E′g_a_τ for that gene is given by:


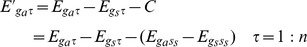
(3)

From Eq. (3), it is clear that all relative expression levels for a seed gene are zero and all genes have a zero relative expression level on the seed time point s_s_.

The consequences of removing non-coherent time points according to the threshold value are illustrated in Figure 3 (b) to Figure 4 (b).

Assuming that *ε* is the threshold of relative expression level for biclustering, then the maximum difference of relative expression level among the genes in a bicluster is 2*ε*. Since an exhaustive enumeration of all combinations of genes and conditions as seeds was performed, two genes, whose maximum difference of relative expression level across a set of time points is less than 2ε, would be clustered into at least one bicluster.

The procedure of the proposed biclustering algorithm is as follows:

1. Pre-process the dataset by filtering out genes that do not show significant variation across conditions or time points. If genes do not show significant differential expression across conditions, they are usually uninteresting and are generally omitted from further analysis. In this study, each gene expression profile is first normalized by z-score (such that the mean µ = 0 and standard deviation σ = 1). Then it is required that for a gene to be included in biclustering analysis, it should satisfy the threshold of: *maximum expression difference across all conditions* >τ where τ = 3ε.
2. For each combination of gene and condition as seed, the relative expression level is computed and a table constructed. Figure 5(a) shows the table constructed for the seed gene “YAR007C” and the seed condition 1. In Figure 5 (a), the first column represents gene. The second column shows conditions where the distance of relative expression between the gene and the *seed gene* is bigger than the threshold ε chosen by the users. In Figure 5(a), the gene YMR078C has “−1” non-coherent conditions, which indicates that all time points of gene YMR078C have a similar relative expression level to the *seed gene* YAR007C.

**Figure 5:**
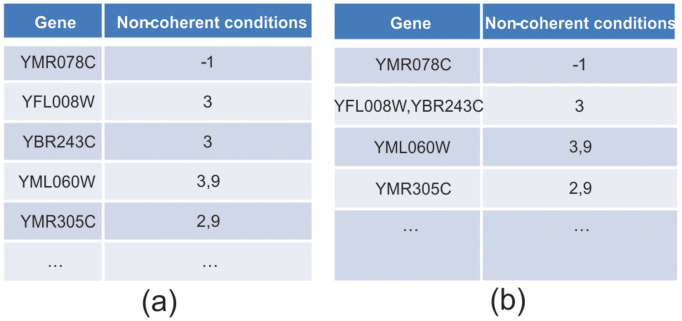
Table constructed for seed gene YAR007C and seed condition 1.
3. Rows with common coherent conditions are identified in the table and grouped together. In this step, all rows that have the same non-coherent conditions are grouped together. The second and third rows (YFL008W and YBR243C) are combined as shown in Figure 5 (b). The table will then be sorted in ascending order of the number of non-coherent conditions. If a row contains more than ***min_gene*** genes, it is considered to be a bicluster and output to the result. This row is then removed from the table. If not, this row is combined into other rows in the table. An example of this latter case is given in Figure 5 (b), where the first row YMR078C is combined into other rows in the table, as shown in Figure 6.

**Figure 6:**
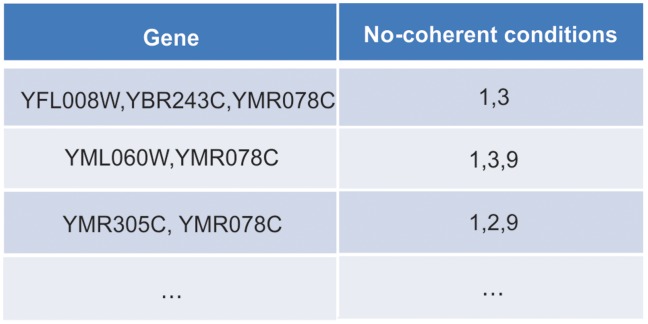
Combined array table.
4. The above procedure is repeated for the subsequent rows. Figure 7 shows how the rows YFL008W, YBR243C andYMR078C are combined with other rows in the table. Since each bicluster must have at least a minimum number of coherent conditions, if a newly combined row has more than (*n* – ***min coherent_condition***) non-coherent conditions, where *n* is the number of conditions in the dataset, then it will not be appended into the table.

**Figure 7:**
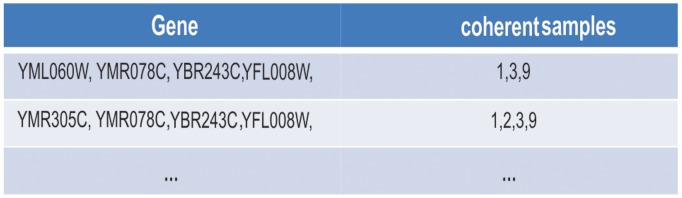
Further combined array table.
5. After the above procedure is repeated for every combination of genes and conditions, all biclusters are obtained.
6. In order to validate the found biclusters, Gene Ontology (GO) is used to test whether these biclusters share the same GO term. This process is done in two phases. First, biclusters that share the same seed gene are aggregated because these genes have similar behaviour across certain conditions or time points. In other words, the biclusters of genes that are generated by the tables having the same seed gene are combined. Second, a statistical test is used to evaluate the aggregated genes to see whether they are enriched by one or more GO terms. The cut-off P value is set to 0.05 in this work.

There are three parameters in the algorithm. The first parameter ε is the maximum relative expression distance on the same time point between two genes in the same bicluster. All gene expression levels are normalized by z-score. As a result, 68% of the relative expression levels are within [−1,1]. Based on the results of this experiment, 0.35 (2ε = 0.7) is considered to be a suitable cut-off value of ε for non-coherent time points.

The second parameter ***min_gene*** represents the minimum number of genes in a bicluster. This parameter cannot be too large, or actual coherent genes may be missed. Five was found to be the most suitable value to use.

The third parameter is ***min_coherent_condition***. This parameter specifies the minimum number of coherent conditions needed in a bicluster and usually depends on the type of microarray data and the applications used.

A flow chat and pseudocode summarizing this algorithm can be found in Figure S1 and Table S1 respectively.

### Test of Statistical Significance of the Detected Biclusters

The normalization step is usually undertaken before microarray data is analysed, to minimize the impact of different experimental conditions. Since the microarray data can be considered to assume a normal distribution [15], z-score is commonly used to normalize microarray data [16]. The z-score can be calculated Eq. (4):


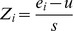
(4)


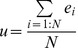
(5)


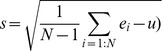
(6)

Where *e_i_* is a gene expression level, *u* and *s* are the mean and standard deviation of the microarray data set respectively. *N* is the total number of genes (or probes) in the microarray.

**Figure 8:**
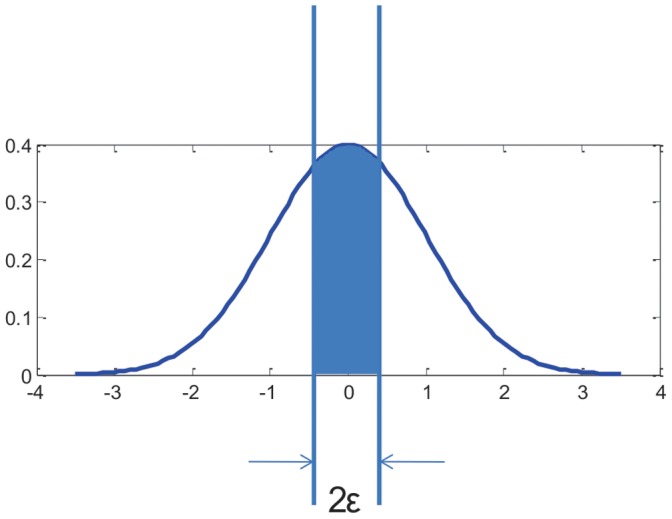
The probability of z-score within 2ε to a specific value.


Since the expression level is normalized by z-score, the probability of a z-score value whose distance to a specific value is smaller than 2ε, is less than normcdf(ε) − normcdf(−ε), as shown in the shaded part of Figure 8. *p* is denoted as the probability of a z-score within the ε distance to a given value.

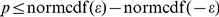
(7)

The *p* value of a gene that has at least the ***min_coherent_condition*** to a seed gene can be represented as a Bernoulli trial:


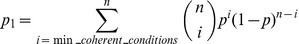
(8)

Where *n* is the total number of time points or conditions. For a total of *m* genes in the study, a bicluster with at least ***min_gene*** genes has the *p* value below.


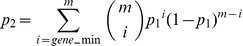
(9)

In this evaluation, ***min_coherent_condition*** was set to 12 and ***min_gene*** was set to 5. For 2,884 genes and 17 time points in the yeast cell cycle expression data, the *p* value to identify a bicluster by chance with ε = 0.35 is less than *p_2_* = 0.00099648. Consequently, the detected biclusters in the yeast cell cycle dataset that satisfy the three conditions, i.e. (1) maximum distance between any two genes is less than ε, (2) the number of conditions is larger than ***min_coherent_condition*** and (3) the number of genes is larger than ***min_gene***, are statistically significant.

### Relationship with H-score in δ-biclustering

The δ-biclustering algorithm of Cheng and Church [1] is a well-known biclustering algorithm. For an microarray expression matrix *m*×*n*, the H-score in δ-biclustering is given by [1]:


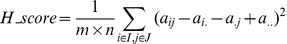
(10)

where I and J are a set of rows and columns respectively, such that |*I*| = *m*, |*J*| = *n*, and *a_ij_* is the expression value in row *i* and column *j*, while *a_i._* is the average expression level of row *i*, *a_j_* is the average expression level of column *j* and *a_.._* is the average expression level of the whole matrix. To intuitively understand the meaning of Eq. 10, the formula can be rewritten as shown in Eq. 11 and 12.


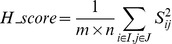
(11)


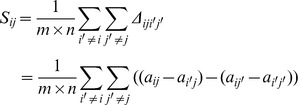
(12)

**Figure 9:**
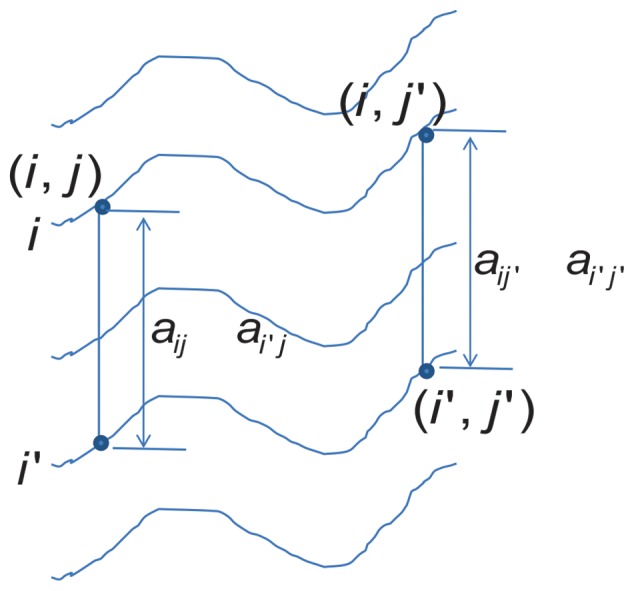
Intuitive illustration of H-score.

It was observed that H-score is the average of score *S_ij_* – that is the average of the differences shown in Eq. 12. Figure 9 shows these differences and clearly indicates that the shift of gene *i′* does not affect *Δ_iji′j′_* and H-score. Therefore, H-score reflects the coherence of an additive bicluster. If (*i*, *j*) are considered to be the seed proposed in this method, the relative expression distance in this method is identical to *Δ_iji′j′_*.

### Implementation of Bicluster Identification Algorithm

The algorithm is implemented in Java computer language. The computer platform is 3.33 GHz CPU and 4G RAM. The operation system is Microsoft Windows XP.

## Results

To evaluate this method, the algorithm was first applied to the well-studied yeast cell cycle time course gene expression data, which has 2,884 genes and 17 time points [17]. Since biclusters that are generated by the same seed gene have the same expression behaviour across most time points or conditions, biclusters that were generated by the same seed gene were aggregated. For simplicity, to the aggregated cluster was also referred as a bicluster, although they may not have similar expression levels in some conditions.

The accuracy of the identified biclusters and the execution time cost was evaluated. The new algorithm was compared to two existing methods: δ-biclustering [1] and pClustering [14]. The biclusters of δ-biclustering were obtained from [17]. With regard to pClustering, the executable code was downloaded from the website in [18] and a range of three parameters (delta i.e. clustering threshold, minimum number of column and minimum number of row) are tested: delta = [5, 10, 20, 30, 40, 50] and minimum number of columns = 10, and minimum number of rows = [5, 20, 30, 40]. delta = 10 and minimum number of rows = 20 were selected because the resultant biclusters are the most similar in number to those identified and reported in [19]. In the new method, ***min_gene*** was set to 5 and ***min_coherent_condition*** was set to 12. Several distances of relative expression level were tested as shown in Table 2. With ε = 0.35, the number of identified biclusters was the most similar to that found in [19].

**Table 2:** The number of biclusters in our comparative study.

method	Our method			δ-cluster	pcluster
	ε = 0.15	ε = 0.35	ε = 0.5		
#cluster	28	102	14	100	132

To test the accuracy of the identified biclusters, the p value was computed using the hypergeometric distribution to compare the detected biclusters to 30 known clusters (or categories) of yeast genes reported by Tavazoie *et al*. [19]. The correspondence plot proposed by Tanay *et al*. [20] illustrates the random chance of genes in the identified bicluster appearing in the putative gene clusters of [19]. The chance is given by the p value computed using Eq. 13.


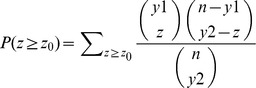
(13)

Where *n* is the total number of genes in the genome; *y_1_* is the number of genes in the putative cluster, *y_2_* is the number of genes in the identified bicluster, *z_0_* is the number of overlapping genes in the two clusters.

**Figure 10:**
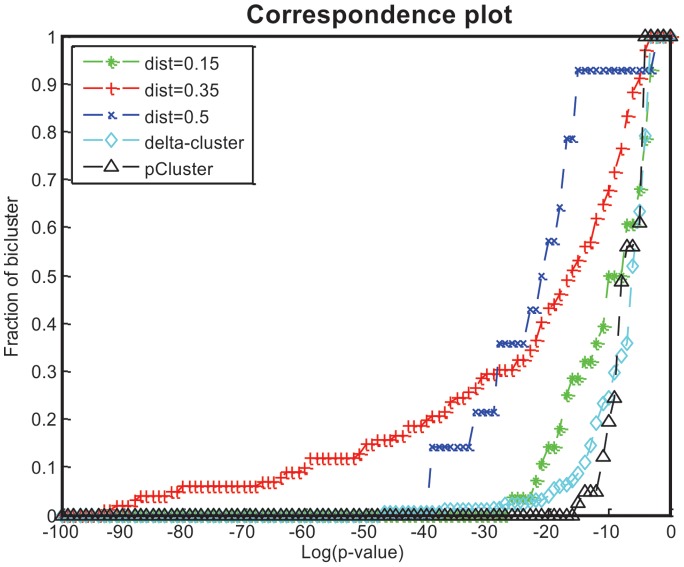
Correspondence plot for the Yeast cell cycle expression data.

Figure 10 shows the correspondence plot for the yeast data, the x-axis represents the p value of found biclusters and the y-axis shows the percentages of found biclusters whose log (p values) are smaller than the value in the x-axis. Since the number of putative clusters in [19] is 30, for each identified bicluster, the smallest p value was selected for the 30 calculated p values. The p values for all identified clusters are sorted in ascending order as shown in Figure 10. The same statistical evaluation was applied to the other two existing algorithms (δ -cluster and pCluster).

As the number of overlapping genes (number of z_0_) increase, the p value of a bicluster decreases. An algorithm is considered better if it has a higher percentage of biclusters with small p value. Figure 10 shows the comparison of this new method with the two existing methods. Most biclusters identified by the new method have relatively smaller p values. Half of the identified biclusters have p value less than 10^−15^. The result of ε = 0.35 has very good overlap with the putative clusters.

For the stem cell data, which has 46 conditions and 21,605 genes and is generated by illumine version2.0, several gene sets that were predicted to be regulated by the same microRNA were identified [21]. For example, genes PIAS3, FCHSD2, MEF2D, SORBS2, ATXN1, and TRIB2 have similar expression profiles and have the same regulating miRNA miR-18a/b. These results also suggest that genes ADAMTS6, LUZP2, PRKAB2, ATXN1, CYFIP2, DCP2, and CCRN4L are regulated by miR-494. These genes have similar expression profiles across 46 adult stem cell lines.

**Figure 11:**
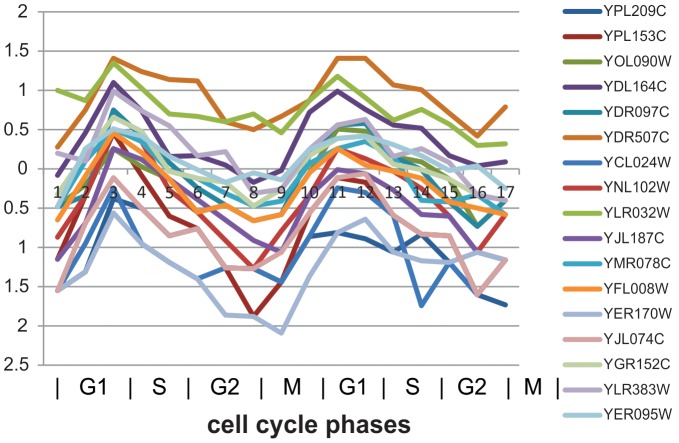
The significance of a bicluster in terms of GO terms.

To evaluate the biological significance of the biclusters in terms of GO (gene ontology), the GO terms that were associated with the highest number of genes in the biclusters were identified. The p value was used to measure significance. Figure 11 shows a biologically significant bicluster of the yeast cell cycle data, which has 17 genes that are associated with GO:0000166 (nucleotide binding): YPL209C, YPL153C, YOL090W, YDL164C, YDR097C, YDR507C, YCL024W, YNL102W, YLR032W, YJL187C, YMR078C, YFL008W, YER170W, YJL074C, YGR152C, YLR383W, YER095W. The p value is 1.4E−04.

A cell cycle consists of four distinct phases: G1(preparation), S (synthesis), G2(interphase), M(mitosis). As shown in Table 3, phase G1 and G2 are the stage of cell growing. Phase S is for DNA replication. Phase M is the last phase to complete cell division.

**Table 3:** Functions in cell cycle.

Phase	G1	S	G2	M
**Biological functions**	Cells increase in size	DNA replication	Cells grow to be ready toenter M phase	Stop growth and ready to complete cell division

Figure 11 shows that the 17 genes related to nucleotide binding are highly expressed in the S (DNA Synthesis) phase. In the M phase, these genes return to their initial expression levels.

**Figure 12:**
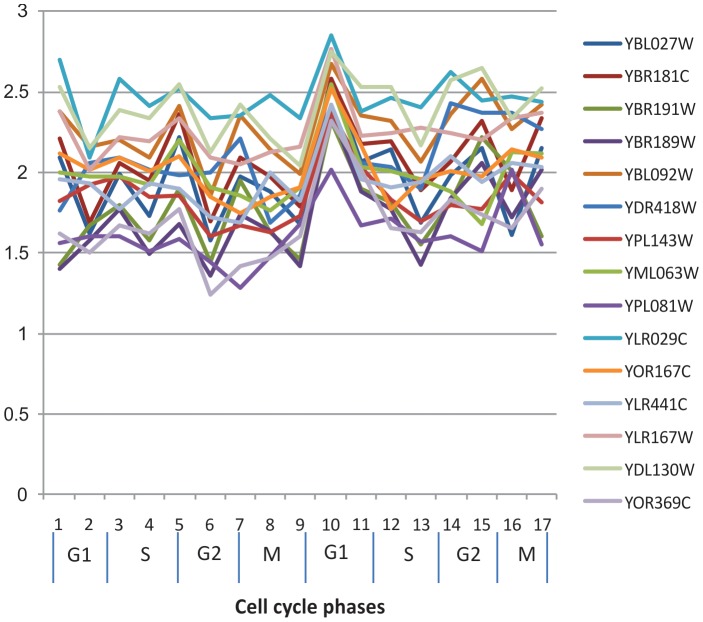
Another significant bicluster in terms of GO.

Figure 12 shows that in one identified bicluster, there are 17 genes YBL027W, YBR181C, YBR191W, YBR189W, YBL090W, YBL092W, YDR418W, YPL143W, YML063W, YPL081W, YLR029C, YOR167C, YLR441C, YLR167W, YDL130W, YOR369C, and YDR494W annotated to GO:0003735 (structural constituent of ribosome). From the expression profiles shown in Figure 12, and in G1 of the second round of cell cycles, these genes are up-regulated sharply because in G (preparation) phase, cells need ribosomes to generate a large number of ribosomal proteins for cell differentiation. This is consistent with the result of [22] which found that a large number of ribosome synthesis factors are up-regulated before cell goes into cell cycle. All the identified biclusters and their associated biological functions are listed in the appendix file Material S1.

### Evaluation of Computation Performance

This method exhaustively enumerates all genes as seed genes. The computation complexity depends on two components: the number of seeds and the computation time for each seed, i.e. *T = n*×*t*, where *T* is the total time, *n* is the number of seeds and *t* is the computation time for each seed. As described in step 1 of “Materials and Method” section, a gene is filtered out when the difference between its maximum and minimum expression values is smaller than τ = 3ε. The greater τ, the more genes are removed from the seed gene list because they do not show enough fluctuation to be included in further analysis, so *n* becomes smaller. However, larger ε increases the similarity tolerance between two gene expression profiles. A larger ε causes the number of genes that are similar to a seed to increase, which results in more distance calculations between genes and the seed gene. The increased number of distance calculations leads to a larger *t*. For low dimensional data, *n* is the dominating factor. For the yeast cell time course data (17-d), the computation time cost reduces when ε is increased from 0.15 to 0.5, which results in τ increasing from 0.45 to 1.5. The decrease in the number of seed genes more than offsets the increase in the number of genes that satisfy the similarity tolerance to a seed gene. Consequently, reducing the number of seeds reduces the total time *T*. However, for very high dimension data (46-d), *T* is dominated by *t* from the distance computation. Hence, increasing ε actually increases the total computation time *T*. Below are listed some empirical results showing computation time costs.

The existing method, pCluster, was run in *cygwin platform*. The new method was tested (with different *ε* value) against the pCluster method on both the yeast cell cycle dataset and the human adult stem cell dataset. Table 4 shows the results. Note that, due to the high dimension and the large number of genes in the human adult stem cell dataset, pCluster yielded a “memory exception” error and failed to run.

**Table 4:** Comparison of time cost.

Time(minutes) dataset	pCluster	Our method		
		ε = 0.15 (τ = 0.45)	ε = 0.35 (τ = 1.05)	ε = 0.5 (τ = 1.5)
Yeast cell cycle (17-d)	0.4333	0.62291664	0.41456667	0.09375
Stem cell (46-d)	N/A	8.586217	11.880575	22.16925

Since the expression data is normalized by z-score, the different data sets are expected to have similar computation complexity. The main cost of computation and memory space depends on the number of rows in the table in step (2). The bigger the table is, the higher the complexity of space and computation. The complexity of the algorithm was analyzed in terms of threshold distance, minimum number of genes, and minimum number of conditions in the biclusters.

**Figure 13:**
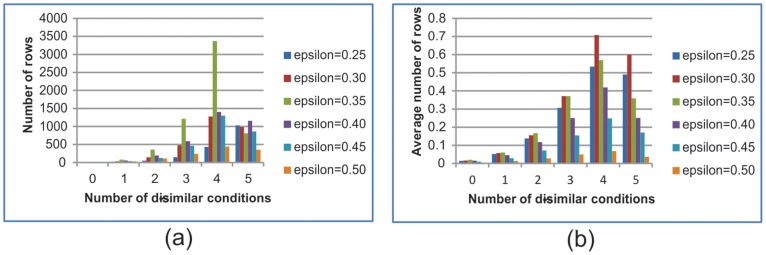
Size of table in terms of distance of relative expression level.

The number of rows in the table was counted in terms of the number of dissimilar conditions: that is, if the number of minimum coherent conditions is 12 and the original expression data has 17 conditions, the number of possible dissimilar conditions should be 0, 1, 2, 3, 4, and 5. Therefore, the number of rows were counted in 0-, 1-, 2-, 3-, 4-, 5- dissimilar conditions. Figure 13 shows the number of rows in the table for the yeast cell cycle data set. Figure 13 (a) shows the maximum number of rows in the table according to the number of dissimilar conditions in terms of distance. Figure 13(b) shows the average number of rows, which is the total number of rows of tables in the whole data set divided by the number of tables or seeds. When the number of dissimilar conditions is four, the table size reaches its maximum. However, in this case, the maximum number of rows in the table is only 3,370, which means our algorithm can be easily be run using a personal computer. From Figure 13(b), it is clear that the average number of rows in the tables was very small, which shows that finding biclusters using the new method does not demand a high cost in time and memory.

**Figure 14:**
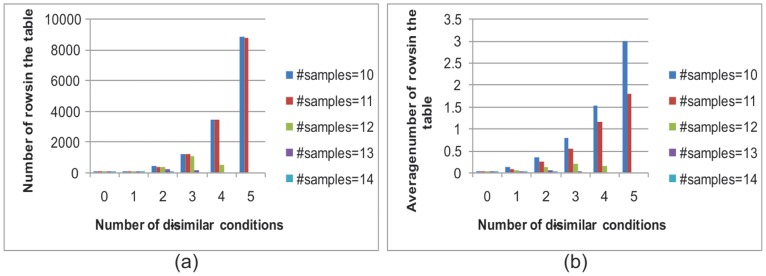
Size of table in terms of minimum number of genes in a bicluster.

Figure 14 shows the size of tables in terms of the minimum number of coherent conditions in each bicluster. Since the yeast cell cycle data has 17 time points, and if the minimum number of coherent conditions is 14, the dissimilar conditions can be 0, 1, 2 and 3. The lower the number of minimum coherent conditions, the more rows the table will have. If the minimum number of coherent conditions in a bicluster is 10, the maximum number of rows in the table is around 8,000, but the average number of rows is only three, confirming that this algorithm can easily run on a personal computer.

## Discussion

Biclustering has been widely researched for many years. However, most biclustering methods are based on heuristic search, which means that the detected biclusters are not optimum. Heuristic search ensures that the biclusters are found in a reasonable time. In this study, all biclusters were exhaustively identified without compromising on quality. The unpromising gene combinations were filtered out at early stage. As a result, the time and space cost of this new method compares favorably to other existing methods.

When the threshold of relative expression level ε is increased, the table used to store all candidate gene combinations is expected to increase in size. However, as ε increases, more candidate genes will have been filtered out in the pre-processing stage, since the threshold τ for filtering out uninteresting gene expression profiles depends on the relative expression level ε (where τ is set to: τ = 3ε). The net result is that the table remains a reasonable size, despite an increase in ε.

In this method, biclusters with more coherent conditions are output first. The biclusters that have fewer coherent conditions are output later. Therefore, it is possible to stop the algorithm at a specific time to get most, if not all, biclusters. For a large microarray data, if one wants to get good biclusters quickly, a time threshold can be set such that only the biclusters that have more coherent conditions are detected.

### Conclusion

This work proposes a new seed-algorithm that performs exhaustive searching of additive biclusters in a large dataset. The central idea of this algorithm is to use all combinations of genes and time points as seeds and create a candidate bicluster table for each seed. The rows in the table are recursively combined. Those rows with more coherent conditions are combined first, and, by doing so, the most potential biclusters are identified and unrelated rows are filtered out at early stage. Although many tables can potentially be created by considering all combinations, most of the tables are very small and have negligible impact on the total search time. In our algorithm, the expression data is normalized by z-score before biclustering. The normalization not only provides a statistical basis for finding significant biclusters, but also reduces the search space (or rows) in the tables. The biclusters detected by this algorithm have better statistical significance than the existing methods. Moreover, the biological significance of the detected biclusters has been biologically confirmed to include genes that have similar expression fluctuation in different cell differentiation stages in the yeast cell cycle dataset.

## Supporting Information

Figure S1 **Flowchart of the algorithm.**
(TIF)

Table S1 **Pseudocode of identifying bi-cluster.**
(DOCX)

Material S1 **In this electronic material S1 we list all biclusters identified by our method.**
(XLSX)
