# Supplementary figures and images for: Seed-Based Biclustering of Gene Expression Data
Source: PLoS One. 2012 Aug 3;7(8):e42431. doi: 10.1371/journal.pone.0042431 (PMC3411756; doi:10.1371/journal.pone.0042431)

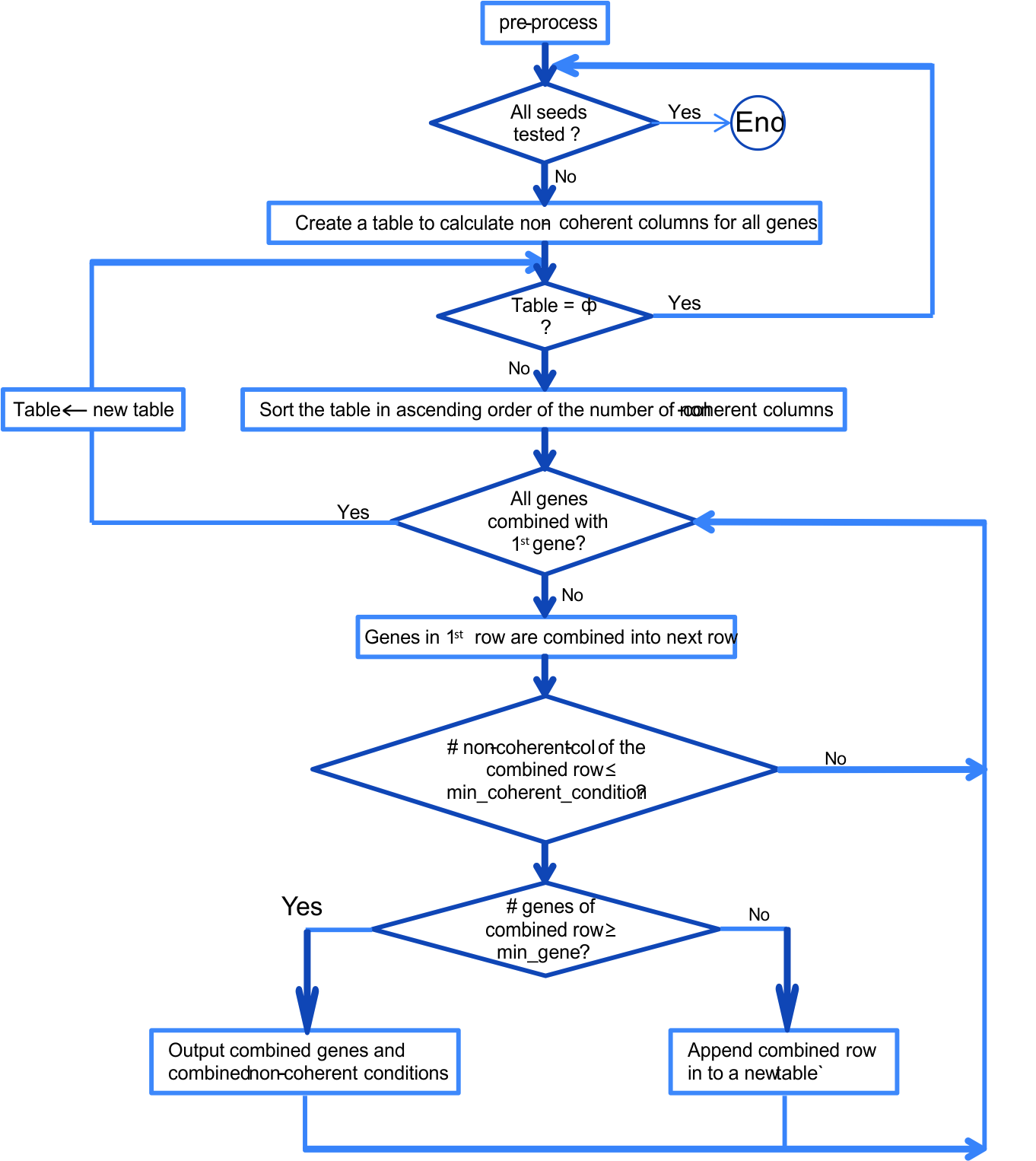

Supplement: Figure S1 — Flowchart of the algorithm. (TIF) [file pone.0042431.s001.tif]
