# Supplementary material for: Seed-Based Biclustering of Gene Expression Data
Source: PLoS One. 2012 Aug 3;7(8):e42431. doi: 10.1371/journal.pone.0042431 (PMC3411756; doi:10.1371/journal.pone.0042431)
Supplement: Table S1 — Pseudocode of identifying bi-cluster. (DOCX) [file pone.0042431.s002.docx]

Table 1S Pseudocode of identifying bi-cluster

| 1. For each seed *s* 2. For each gene *g* 3. *non_coh_col* 🡨 find_coherent_columns(*s*, *g*) 4. *Table* 🡨 *Table*.add{*g*, *non_coh_col*} 5. End For 6. While (*Table* ≠ ф) 7. sort *Table* in ascending order of number of *non_coh_col* 8. *genes* 🡨 *Table*{*row_no* = 1}.*g* 9. *cols* 🡨 *Table*{*row_no* = 1}.*non_coh_col* 10. *new_table* 🡨 *ф* 11. For (*row_no* = 2 : nrow*(Table)*) 12. *combined_genes* 🡨 *Table*{*row_no*}.*g* U *genes* 13. *combined_col* 🡨 *Table*{*row_no*}.*non_coh_col U cols* 14. if (# *combined_col* ≤ ***min_coherent_condition***) 15. if (# *combined_gene* ≥ ***min_gene***) 16. output {*combined_genes, combined_col*} 17. else 18. *new_table* 🡨 *new_table*.add(*combined_genes, combined_col*) 19. endif 20. endif 21. End For 22. *table* 🡨 *new_table* 23. End while 24. End For |
| --- |

In line 1, seed *s* is a combination of a gene and a condition. Line 2-5 creates a table which contains all genes and their non-coherent conditions in terms of seed *s*. Line 6-23 finds all bi-clusters in terms of seed s. Line 7 sorts the created table in order of the number of non-coherent conditions. As shown in Figure 5, rows that have fewer non-coherent columns are listed on the top of the table. Line 8 and 9 work with code Lines 10-22 to select the top row in the table and combine it with all other relevant rows in the table. Line 12 and 13 sort the current row’s genes and non-coherent conditions into variables combined_genes and combined_col. In line 14- 20, if the combined_col has more than min_coherent_condition, the combined row is filtered out.It will not be considered any more. Otherwise, if the number of genes in the row is more than min_gene, the genes and conditions are outputted because it is identified as a bicluster according the parameters. If the number of genes has not reached the min_gene, a new row must be further tested and put it into the new_table. Line 22 assigns new_table to the table for further combination.
